# Nephrologist referrals of older patients with chronic kidney disease in Singapore: a cross-sectional study

**DOI:** 10.3399/BJGPO.2021.0155

**Published:** 2022-08-10

**Authors:** Wei Beng Tan, Anna Szücs, Sarah M Burkill, Ong Shih Hui, Doris Young, Goh Lay Hoon

**Affiliations:** 1 National University Polyclinics, National University Health System, Singapore, Singapore; 2 Division of Family Medicine, Department of Medicine, National University of Singapore, Singapore, Singapore; 3 Saw Swee Hock School of Public Health, National University Singapore, Singapore, Singapore; 4 Regional Health System Office, National University Health System, Singapore, Singapore; 5 Division of Family Medicine, Yong Loo Lin School of Medicine, National University Singapore, Singapore, Singapore

**Keywords:** renal insufficiency, chronic, older patients, primary healthcare, referral, nephrology, family medicine, population health, general practive

## Abstract

**Background:**

Chronic kidney disease (CKD) is common in the older population. By 2035, approximately one-quarter of Singapore residents are expected to have CKD. Many of these patients are not referred to nephrologists.

**Aim:**

To compare the characteristics of older patients (aged ≥65 years) with CKD stage ≥3B in the referral and non-referral groups.

**Design & settings:**

A cross-sectional study in the primary care organisation National University Polyclinics (NUP), Singapore.

**Method:**

Retrospective data were extracted from the electronic health records of patients with CKD (aged ≥65 years) with CKD stage ≥3B.

**Results:**

From 1 January–31 December 2018, a total of 1536 patients aged ≥65 years were diagnosed with CKD stage ≥3B (non-referral group = 1179 versus referral group = 357). The mean patient age in the non-referral group (78.4 years) was older than that in the referral group (75.9 years) (*P*<0.001). Indian older patients were referred more compared with their Chinese counterparts (*P* = 0.008). The non-referral group was prescribed significantly less fibrate, statins, insulin, sulfonylureas, dipeptidyl peptidase-4 (DPP4) inhibitors, and antiplatelet than the referral group (*P*<0.05), but only the difference in fibrates remained significant on subsequent multivariate analysis.

**Conclusion:**

This study demonstrates that there is a considerable number of older patients with CKD exclusively managed in the primary care setting (*n* = 1179) and that referrals primarily depend on demographic factors, namely age and ethnic group, rather than medical determinants of CKD severity or case complexity.

## How this fits in

This study reflects a vital role of family physicians in managing older patients with CKD and highlights the need to review the referral process among this diverse group of patients. Multidisciplinary collaboration between family physicians and nephrologists is recommended to refine the referral criteria to determine who truly needs early referrals to a nephrologist, and to develop guidelines to optimise the primary care management and monitoring of patients with CKD, especially of those who are not referred and treated conservatively.

## Introduction

CKD is a common presentations among the older population in the primary care setting.^1–3^ In the US, the Third National Health and Nutrition Examination Survey (NHANES III) conducted between 1988 and 1994 demonstrated that 7.6% of individuals aged 60–69 years and 25.9% of those aged at least 75 years had a glomerular filtration rate (GFR) of 15–60 ml per minute per 1.73 m^2^ compared with only 1.8% of those aged 40–59 years and 0.2% of those aged <40 years^4^. In France, an epidemiological survey of the Île-de-France area showed that the incidence rate among patients aged >75 years was almost seven times that of patients aged 20–39 years (619 versus 92 new cases per million population) and more than double that of patients aged 40–59 years (619 versus 264 new cases per million population).^5^ In Singapore, it is projected that from 2007–2035, the number of residents with CKD will increase from 316 521 to 887 870, indicating an increase in prevalence from 12.2% to 24.3%.^6^ By 2035, approximately one-quarter of Singapore residents are expected to have CKD. This trend will likely affect how patients with CKD are managed in the primary care community.^6^

CKD management has become part of multi-chronic disease management for family physicians in Singapore. With the introduction of CKD classification by Kidney Disease: Improving Global Outcomes (KDIGO),^7^ the resultant increasing awareness of CKD in primary care settings had a significant impact on referral patterns to renal medical services with increased referral rates, as reported in Boston, US^8^ and Brisbane, Australia.^9^ However, comparative studies contrasting the characteristics of older patients with CKD between referral and non-referral groups in the primary care setting are lacking.

In Singapore, NUP is the public primary care provider of the western cluster healthcare system known as the National University Health System (NUHS). It offers subsidised family medical care services to communities in western Singapore. In April 2017, the Holistic Approach in Lowering and Tracking CKD (HALT-CKD) programme was launched by the Ministry of Health, aiming to do the following: (1) recruit and track all patients with stage 1–4 CKD from any cause; (2) slow down CKD progression with control of risk factors and renin-angiotensin system (RAS) blockade in all stages of CKD; and (3) encourage shared-care collaboration between primary health care and nephrologists at stage 3B–4 CKD.^10^ This programme recommends that patients with CKD stage ≥3B be referred to nephrologists at secondary care hospitals. This is to provide the patients with early access to further investigations by nephrologists and preparation for renal replacement therapies to reduce morbidity, mortality, and hospitalisation rates.^11,12^ However, many older patients are managed by the primary care team and are not referred to renal physicians. The factors contributing to the referral preferences are not well studied.

The objective of the study was to compare the characteristics of older patients (aged ≥65 years) with CKD 3B, 4, and 5 who were referred to nephrologists with those who were not referred at NUP in Singapore from 1 January 2018–31 December 2018. The null hypothesis was that there was no significant difference in CKD severity, sociodemographic factors, comorbidities, or medication between the referral and the non-referral groups.

## Method

’CKD’ was defined as per the KDIGO classification,^7^ and ‘older patients’ as those aged ≥65 years.^13^ Retrospective data were collected on all older patients with CKD stage ≥3B at five NUP polyclinics (Bukit Batok, Choa Chu Kang, Clementi, Jurong, and Pioneer) between 1 January 2018 and 31 December 2018, using the NUP electronic record system. As the CKD status can be confirmed only with two consecutive estimated GFRs (eGFR ml/min/1.73 m^2^) 90 days apart, data extraction was performed from 1 October 2017 until 31 December 2018. Estimated glomerular filtration rate (eGFR) results are reported based on the CKD Epidemiology Collaboration (CKD-EPI) equation: serum creatinine (µmol/l), age (years), sex. Albuminuria and proteinuria categories were defined based on the albumin-to-creatinine ratio (ACR), and when not available, based on the protein-to-creatinine ratio (PCR), following the cut-off values of the KDIGO classification^7^: ACR <3 mg/mmol or PCR <15 mg/mmol, which is normal to mildly increased; ACR of 3–30 mg/mmol or PCR of 15–50 mg/mmol, which is moderately increased; and ACR >300 mg/mmol or PCR >50 mg/mmol, which is severely increased.

The inclusion criteria were as follows:

1. Patients aged ≥65 years.
2. The stages of CKD in patients were confirmed when there were two eGFRs in ml/min/1.73 m^2^ 90 days apart, defined as:

- Stage 3B (eGFR: 30–44 ml/min/1.73 m^2^)
- Stage 4 (eGFR: 15–29 ml/min/1.73 m^2^)
- Stage 5 (eGFR:<15 ml/min/1.73 m^2^).

The exclusion criteria were as follows:

1. Patients with stages 1–3A CKD or an unknown CKD stage or status (lack of two consecutive eGFR results at least 90 days apart).

The following data were extracted from the electronic records for all eligible patients: demographics (age, sex, ethnic group, and smoking status), comorbidities (diabetes mellitus, hypertension, dyslipidaemia, ischaemic heart disease, cerebrovascular disease, and peripheral vascular disease), and medications as of the date where CKD status was established (angiotensin-converting enzyme inhibitors [ACEI], angiotensin receptor blockers (ARB), statins, fibrates, biguanides, sulfonylureas, loop or potassium-sparing diuretics, insulin, and antiplatelets).

The characteristics of patients were compared between those who were referred by the family physicians to nephrologists and those who were not.

### Statistical analysis

Statistical analysis was performed using Stata (version 16.0) and R (version 3.6.1). χ^2^ tests were used for categorical variables; *t*-tests for continuous variables in bivariate comparisons between referral and non-referral groups; and two-way ANOVA to assess the mean number of comorbid diseases by age group in referral versus non-referral groups. In a further confirmatory analysis, a stepwise logistic regression was run predicting referral with a first model containing only CKD status and albuminuria and proteinuria, as these two factors determine prognosis in the KDIGO classification^7^. The subsequent models included variables that were significant in the bivariate analysis, entered hierarchically by category (sociodemographic factors, comorbidities, and medication). Missing values were handled by listwise deletion (complete-case analysis was performed).

## Results

From 1 January–31 December 2018, a total of 1536 patients aged ≥65 years were diagnosed with CKD stage ≥3B (Table 1). There were 1179 patients in the non-referral group and 357 in the referral group. Data on blood pressure was missing in four participants (0.26% of the total sample). HbA1c data were missing in 50 out of 1097 patients with diabetes mellitus (4.56%). Thirty-three patients (2.15% of the total sample) had a PCR but no ACR value: 19 patients (1.61%) of the non-referral group and 14 patients (3,92%) of the referral group. Values for both ACR and PCR were missing in 183 patients (11.91% of the total sample), 154 from the non-referral group (13.06%) and 29 from the referral group (8.12%).

**Table 1.** Patient demographics

	Non-referral group	Referral group	*P* value
Sex*, n* (%)			
Female	621 (52.67)	188 (52.66)	
Male	558 (47.33)	169 (47.34)	0.997
**Age group*, n* (%)**			
65–69	167 (14.16)	77 (21.57)	
70–74	226 (19.17)	74 (20.73)	
75–79	258 (21.88)	107 (29.97)	
80–84	267 (22.65)	52 (14.57)	
85–89	177 (15.01)	35 (9.80)	
≥90	84 (7.12)	12 (3.36)	<0.001
**Mean age, years (SD)**	78.35 (7.51)	75.88 (6.83)	<0.001
**Ethnic group*, n* (%)**			
Chinese	907 (76.93)	250 (70.03)	
Indian	48 (4.07)	21 (5.88)	
Malay	204 (17.30)	72 (20.17)	
Others	20 (1.70)	14 (3.92)	0.017
**Smoking status*, n* (%)**			
Formerly smoked	39 (3.31)	16 (4.48)	
Never smoked	1089 (92.37)	317 (88.80)	
Currently smokes	51 (4.33)	24 (6.72)	0.097

*P* value is taken from χ^2^ test for categorical, and *t*-test for continuous variables

The bivariate analysis indicated a significant difference in age between those who were not referred and those who were referred (means: 78.4 years versus 75.9 years), regardless of age being coded as a continuous or a categorical variable (both *P*<0.001). The χ^2^ test was significant with respect to ethnic groups (*P* = 0.017; Table 1). There was no significant difference between the non-referral and referral groups on CKD severity (Table 2) or comorbidities (Table 3). The groups differed for the following medications: fibrates, statins, insulin, sulfonylureas, DPP-4 inhibitors, and antiplatelets (Table 4).

**Table 2.** Comparison of indicators of CKD severity (CKD status and albuminuria and proteinuria) of non-referral and referral groups

	**Non-referral group, *n* (%**)	Referral group*, n* (%)	*P* value
**CKD status**			
CKD 3B	856 (72.60)	249 (69.75)	
CKD 4	279 (23.66)	96 (26.89)	
CKD 5	44 (3.73)	12 (3.36)	0.453
**Albuminuria and proteinuria^a^**			
Normal to mildly increased	290 (28.29)	97 (29.57)	
Moderately increased	402 (39.22)	109 (33.23)	
Severely increased	333 (32.49)	122 (26.8137.20)	0.126

*P* value is taken from χ^2^ tests.^a^Albuminuria and proteinuria data (albumin-to-creatinine ratio [ACR] or protein-to-creatinine ratio [PCR]) were available for 1353 patients, 1025 in the non-referral and 328 in the referral group. CKD = chronic kidney disease

**Table 3.** Comparison of patient comorbidities between non-referral and referral groups

	Non-referral group	Referral group	*P* value
**Diagnoses** ***, n*** **(%)**			
Diabetes mellitus	837 (70.99)	260 (72.83)	0.544
Hypertension	1165 (98.81)	348 (97.48)	0.117
Hyperlipidaemia	1155 (98.00)	351 (98.30)	0.837
Gout	233 (19.80)	65 (18.21)	0.566
Ischaemic heart disease	296 (25.11)	91 (25.49)	0.939
Peripheral vascular disease	93 (7.89)	25 (7.00)	0.662
Stroke	268 (22.73)	72 (20.17)	0.343
Dementia	93 (7.89)	25 (7.00)	0.662
**Mean number of comorbid diagnoses (SD)**	4.51 (1.01)	4.46 (1.00)	0.443
**Mean number of comorbid diagnoses by age group (SD)**			
65–69	4.54 (1.01)	4.34 (1.01)	0.136
70–74	4.48 (1.01)	4.61 (1.01)	0.352
75–79	4.55 (1.01)	4.41 (1.01)	0.218
80–84	4.52 (1.01)	4.31 (1.01)	0.172
85–89	4.50 (1.01)	4.71 (1.01)	0.258
≥90	4.39 (1.01)	4.83 (1.01)	0.158
**Mean blood pressure (SD)** ^**a**^			
Systolic	133.33 (16.24)	134.17 (14.54)	0.357
Diastolic	67.71 (9.41)	68.55 (8.94)	0.125
**Mean HbA1c (SD)** ^**b**^	7.35 (1.34)	7.46 (1.46)	0.261

*P* value is taken from χ^2^ test for categorical, unpaired *t*-test for continuous variables, and two-way ANOVA for the mean number of comorbid diagnoses by age group.^a^Blood pressure data available for 1532 patients: 1177 in non-referral and 355 in referral group; ^b^HbA1c data available for 1047/1097 patients with diabetes mellitus: 795 in non-referral and 252 in referral group

**Table 4.** Comparisons of patient medications between non-referral and referral groups

	Non-referral group, *n* (%)	Referral group, *n* (%)	*P* value
**Drugs**			
Fibrates	55 (4.66)	28 (7.84)	0.020
Statins	959 (81.34)	311 (87.12)	0.012
Insulin	187 (15.86)	76 (21.29)	0.017
Biguanides	281 (23.83)	88 (24.65)	0.752
Sulfonylureas	331 (28.07)	123 (34.45)	0.021
DPP4	184 (15.61)	79 (22.13)	0.004
ARB	435 (36.90)	144 (40.34)	0.24
ACEI	363 (30.79)	107 (29.97)	0.769
CCB	707 (59.97)	223 (62.46)	0.433
Loop diuretics	302 (25.61)	95 (26.61)	0.707
Potassium-sparing diuretics	28 (2.37)	5 (1.40)	0.266
Alpha-blockers	42 (3.56)	6 (1.68)	0.073
Beta-blockers	490 (41.56)	157 (43.98)	0.418
Antiplatelets	408 (34.61)	147 (41.18)	0.024

*P* value is taken from χ^2^ tests. ACEI = angiotensin-converting enzyme inhibitors. ARB = angiotensin receptor blockers. CCB = calcium channel blockers. DPP4 = dipeptidyl-peptidase 4 inhibitors.

Logistic regression did not find significant associations between referrals and CKD status or albuminuria and proteinuria (Table 5). In model 2, additionally testing sociodemographic variables, the older age groups, namely 80–84 years (odds ratio [OR] 0.43, 95% confidence interval [CI] = 0.28 to 0.67], *P*<0.001), 85–89 years (OR 0.49, 95% CI = 0.30 to 0.79, *P* = 0.003), and ≥90 years (OR 0.41, 95% CI = 0.20 to 0.84, *P* = 0.015) were less likely to be referred than the 65–69 years group. With respect to ethnic group, both Indians and the ethnicities grouped under 'Others' had significantly higher odds to be referred than Chinese patients (OR 2.18, 95% CI = 1.23 to 3.86, *P* = 0.008 and OR 2.74, 95% CI = 1.32 to 5.70, *P* = 0.007, respectively). These effects of age and ethnic group remained significant in model 3, where medications were also included. However, of the drugs that were significant in the bivariate analysis, only fibrates remained weakly significant in the multivariate analysis (OR 1.69, 95% CI = 1.00 to 2.83, *P* = 0.049).

**Table 5.** Logistic regression predicting likelihood of referral with all variables significant in the bivariate analysis

	Model 1		Model 2		Model 3	
	OR (95% CI)	*P* value	OR (95% CI)	*P* value	OR (95% CI)	*P* value
**CKD severity**						
**CKD status**						
CKD_catG3b	Ref.		Ref.		Ref.	
CKD_catG4	1.23 (0.92 to 1.63)	0.162	1.30 (0.97 to 1.74)	0.081	1.30 (0.97 to 1.74)	0.085
CKD_catG5	1.02 (0.53 to 2.00)	0.943	0.99 (0.50 to 1.96)	0.977	0.98 (0.49 to 1.96)	0.961
**Albuminuria and proteinuria**						
Normal to mildly increased	Ref.		Ref.		Ref.	
Moderately increased	0.82 (0.60 to 1.12)	0.201	0.89 (0.64 to 1.22)	0.243	0.87 (0.63 to 1.21)	0.410
Severely increased	1.11 (0.81 to 1.51)	0.518	1.18 (0.86 to 1.62)	0.318	1.14 (0.83 to 1.58)	0.417
**Sociodemographics**						
**Age group, years**						
65–69			Ref.		Ref.	
70–74			0.75 (0.51 to 1.13)	0.168	0.77 (0.52 to 1.16)	0.208
75–79			0.97 (0.66 to 1.41)	0.856	1.01 (0.69 to 1.48)	0.962
80–84			0.43 (0.28 to 0.67)	<0.001^a^	0.47 (0.30 to 0.73)	<0.001^a^
85–89			0.49 (0.30 to 0.79)	0.003^b^	0.56 (0.34 to 0.91)	0.020^c^
≥90			0.41 (0.20 to 0.84)	0.015^c^	0.48 (0.23 to 0.99)	0.046^c^
**Ethnic group**						
Chinese			Ref.		Ref.	
Indian			2.18 (1.23 to 3.86)	0.008^b^	2.07 (1.16 to 3.70)	0.014^c^
Malay			1.23 (0.88 to 1.70)	0.223	1.21 (0.87 to 1.68)	0.268
Others			2.74 (1.32 to 5.70)	0.007^b^	2.82 (1.35 to 5.89)	0.006^b^
**Medication**						
Fibrates					1.69 (1.00 to 2.83)	0.049
Statins					1.35 (0.91 to 2.00)	0.141
Insulin					1.01 (0.72 to 1.43)	0.935
Sulfonylureas					1.15 (0.85 to 1.54)	0.365
DPP4					1.20 (0.85 to 1.71)	0.295
Antiplatelets					1.23 (0.94 to 1.60)	0.131

Results indicate the odds of being referred. ^a^ *P*<0.001; ^b^ *P*<0.01; ^c^ *P*<0.05. CKD = chronic kidney disease. DPP4 = dipeptidyl-peptidase 4 inhibitors.

## Discussion

### Summary

This study demonstrates that primary care services, such as NUP in western Singapore, manage a considerable number of older patients with CKD instead of referring them to nephrologists (*n* = 1179, 76.76%). It is noteworthy that the HALT-CKD programme’s recommendation includes referral for patients with stage 3B CKD, which contrasts with the KDIGO guidelines that recommend referral to kidney specialists for patients who have stage 4 or 5 CKD.^14^ However, the present study showed that CKD severity or comorbidities may not contribute to patient referrals.

On the other hand, the study found that patients aged >80 years were less likely to be referred. Additionally, among ethnic groups, Indian patients were more likely to be referred than Chinese patients. Other studies also highlighted age and ethnic groups as possible implicit factors affecting treatment processes in healthcare management.^15,16^ Differences in age groups and ethnic groups may suggest underlying sociocultural factors affecting patients’ preferences and the family physician’s beliefs, attitude, and understanding in managing older patients with CKD. In the case of age, the non-referral could arise from a shared decision made between the family physicians and the older patients with CKD to favour continued management in the community. Furthermore, language barriers may influence referrals, as most physicians at NUP are English-speaking and from a Chinese ethnic group. Although English is the main language, the heterogenous sociocultural backgrounds of Singaporean patients likely played a role in the dynamic interaction among older patients, their caregivers, and family physicians.

Bivariate analysis also suggested that more referral group patients were taking fibrates, statins, insulin, sulfonylureas, DPP-4 inhibitors, and antiplatelets than the non-referral group. However, only a weak difference in fibrates remained in the multivariate analysis. It is, therefore, possible that the initial bivariate differences found in medications were confounded by age. As the referral group of patients with CKD was comparatively younger than the non-referral group, their chronic diseases were likely to be treated with more aggressive treatments.

### Strengths and limitations

This study is one of the first retrospective cross-sectional studies to investigate older patients with CKD enrolled in a single primary care organisation with a diverse mix of ethnic groups in Southeast Asia. It highlights the role of age and ethnic group in the decision to refer older patients with CKD to a nephrologist, demonstrating the real-life management of older patients with CKD patients in the community.

Further qualitative studies to account for these differences are warranted to gain insights into the reasons underlying the decisions in the referral process, involving primary care physicians, patients, and their families.

This study did not investigate whether the non-referral group could be further divided into subgroups; that is, those who were already followed by a nephrologist before the study period, those who declined referral to a nephrologist, those who defaulted to hospital follow-ups, and those who were discharged from the hospital and/or were treated at private health facilities. While duplicate follow-ups for CKD by NUP and nephrologists are likely avoided by most patients owing to unnecessary costs, it cannot be excluded that more patients from older age groups have seen a nephrologist in the past, as they had likely had CKD for a longer time than the younger patients. Data collection was done in a cross-sectional manner over the course of 1 year and did not contain outcome data. Future research into outcomes for those who are referred and those who are not referred would provide useful insights. Changes in GFR or albuminuria and proteinuria were not studied. Finally, data on both ACR and PCR were missing in approximately one-tenth of the sample, which reduced the power of the analyses to detect differences in albuminuria and proteinuria.

### Comparison with existing literature

Torreggiani *et al* showed that 70% of their hospital renal clinic outpatients were aged ≥60 years and 25% were aged ≥80 years.^17^ However, in their study, only approximately 50% of these patients had CKD stage ≥3B CKD.^17^ This contrasts with the present study population, where the NUP local guidelines do not recommend referral to a nephrologist below stage 3B. Torreggiani *et al* also revealed that the majority of the causes of CKD are multifactorial diseases, nephroangiosclerosis, and diabetes-associated kidney disease, particularly the variant with low proteinuria (diabetes-vascular), which increases with age.^17^ This variant accounts for over 80% of the diagnoses in patients aged ≥80 years. Unlike outpatient nephrologist clinics, where causes of kidney diseases can be confirmed using hospital diagnostic support, such as renal biopsy and imaging, almost all patients with CKD in primary care in Singapore are diagnosed biochemically and with an ultrasound scan to rule out underlying obstructive nephropathy.

Conservative management of CKD is gradually being recognised as a viable therapeutic alternative for patients with advanced CKD.^18,19^ Early detection leads to early management of the associated risk factors to optimise medical care in the older population.^5,6^ Most of these risk factors can be identified and managed in the primary care setting. In the UK, McClure *et al* retrospectively studied 124 patients in the hospital setting who were aged ≥80 years and had stage 4 (115 patients) or 5 (nine patients) CKD.^20^ Forty-seven per cent of their patients were discharged to primary care with median time to death being 3.57 years versus 2.66 years for those who remained in the nephrologist follow-up. This study suggests that the majority of older patients can be safely and appropriately managed in the primary care setting.

However, there are reported challenges that affect the delivery of CKD care in primary care.^21^ These include suboptimal screening or monitoring of CKD risk factors,^22,23^ infrequent discussions between providers and patients regarding CKD,^20^ suboptimal albuminuria testing,^23,24^ suboptimal blood pressure control,^23^ suboptimal renin-angiotensin blockade in patients with CKD with proteinuria,^25,26^ limited knowledge of CKD risk factors,^27,28^ and poor awareness of Kidney Disease Outcomes Quality Initiative (KDOQI) clinical practice guidelines.^29–32^ The present study shows that 381 (32.31%) of patients in the non-referral group and 106 (29.69%) of patients in the referral group were not prescribed a reno-protective ACEI or ARB. This may be explained by the average blood pressure readings of 133/67 mmHg and 134/68 mmHg, respectively, in the two groups (Table 3). Lowering blood pressure further could be contraindicated in these older groups of patients. However, among the older patients with CKD with diabetes, the diabetic control was good, with both groups showing HbA1c <8.0% (Table 3).

### Implications for practice

This study highlights that family physicians at NUP managed 76.76% of the total older patients with CKD with stage 3B, 4, and 5 diseases. This reflects a vital role of family physicians in managing older people with severe CKD. This study highlights the need to review the referral process in this diverse group of patients and to better understand the role of sociodemographic factors in this context. Multidisciplinary collaboration is recommended between family physicians and nephrologists to refine the referral criteria to detect patients who truly need early referrals to nephrologists. It is also recommended that guidelines should be developed to optimise primary care management and monitoring of patients with CKD, especially for those who are not referred and treated conservatively.

## Data

As the identities of the participants in this study need to be protected, the dataset this study relies on cannot be made publicly available.
